# Implementation of Intraoperative Ultrasound Localization for Breast-Conserving Surgery in a Large, Integrated Health Care System is Feasible and Effective

**DOI:** 10.1245/s10434-021-10454-8

**Published:** 2021-08-26

**Authors:** Jeffery M. Chakedis, Annie Tang, Gillian E. Kuehner, Brooke Vuong, Liisa L. Lyon, Lucinda A. Romero, Benjamin M. Raber, Melinda M. Mortenson, Veronica C. Shim, Nicole M. Datrice-Hill, Jennifer R. McEvoy, Vignesh A. Arasu, Dorota J. Wisner, Sharon B. Chang, The Permanente Medical Group Breast Research Collaborative

**Affiliations:** 1 Department of General Surgery and Radiology, The Permanente Medical Group (TPMG), Oakland, CA USA; 2 Department of Surgery, University of California San Francisco, East Bay-Highland Hospital, Oakland, CA USA; 3 Kaiser Permanente Northern California Division of Research, Oakland, CA USA; 4 Department of Surgery, Kaiser Permanente Fremont Medical Center, Fremont, CA USA

## Abstract

**Background:**

Intraoperative ultrasound (IUS) localization for breast cancer is a noninvasive localization technique. In 2015, an IUS program for breast-conserving surgery (BCS) was initiated in a large, integrated health care system. This study evaluated the clinical results of IUS implementation.

**Methods:**

The study identified breast cancer patients with BCS from 1 January to 31 October 2015 and from 1 January to 31 October 2019. Clinicopathologic characteristics were collected, and localization types were categorized. Clinical outcomes were analyzed, including localization use, surgeon adoption of IUS, day-of-surgery intervals, and re-excision rates. Multivariate logistic regression analysis was performed to evaluate predictors of re-excision.

**Results:**

The number of BCS procedures increased 23%, from 1815 procedures in 2015 to 2226 procedures in 2019. The IUS rate increased from 4% of lumpectomies (*n* = 79) in 2015 to 28% of lumpectomies (*n* = 632) in 2019 (*p* < 0.001). Surgeons using IUS increased from 6% (5 of 88 surgeons) in 2015 to 70% (42 of 60 surgeons) in 2019. In 2019, 76% of IUS surgeons performed at least 25% of lumpectomies with IUS. The mean time from admission to incision was shorter with IUS or seed localization than with wire localization (202 min with IUS, 201 with seed localization, 262 with wire localization in 2019; *p* < 0.001). The IUS re-excision rates were lower than for other localization techniques (13.6%, vs 19.6% for seed localization and 24.7% for wire localization in 2019; *p* = 0.006), and IUS predicted lower re-excision rates in a multivariable model (odds ratio [OR], 0.59).

**Conclusions:**

In a high-volume integrated health system, IUS was adopted for BCS by a majority of surgeons. The use of IUS decreased the time from admission to incision compared with wire localization, and decreased re-excision rates compared with other localization techniques.

**Supplementary Information:**

The online version contains supplementary material available at 10.1245/s10434-021-10454-8.

Breast-conserving surgery for non-palpable breast cancers relies on accurate localization of the tumor for complete surgical excision. Localization techniques include invasive procedures, such as placement of wires or radioactive/nonradioactive seeds, and noninvasive methods, such as intraoperative ultrasound (IUS).^1–3^ Wire localization is the most commonly used technique, but it requires a separate procedure by the breast-imaging team, which can increase patient anxiety and create challenges for surgery scheduling.^4^

Non-radioactive seeds can be placed before the day of surgery to decouple cancer localization from the day of surgery, improving patient experience and facilitating surgery scheduling.^5^ However, the disposables and consoles are expensive, and placement still requires an invasive procedure by a breast radiologist.

In contrast, IUS does not require an additional invasive procedure and uses ultrasound (US) units, which many operating rooms and surgery offices already own. The benefits include improved patient satisfaction, ease of scheduling, decreased radiology utilization, and decreased costs.^6–14^ Importantly, IUS can be used for small, non-palpable breast cancers, which comprise the bulk of early-stage disease.^15,16^

A growing number of surgeons use point-of-care US in training and practice, but IUS has not been used as much as other wire- or seed-based methods. This may be due to concerns about the investment of time and money into learning a new technique, although the learning curve for IUS is relatively short, requiring only a few operations.^13^

Nearly all published reports of IUS are single-institution studies, and a method of implementing an IUS program across a large group of surgeons and variety of surgery practices has not been described to our knowledge.

In 2015, the Permanente Medical Group (TPMG), a large, integrated health care system, launched a pilot IUS project demonstrating rapid adoption of IUS with favorable outcomes.^17^ The pilot project was expanded to a program throughout Kaiser Permanente Northern California (KPNC) hospitals and provided support for surgeons to acquire training and equipment as needed. In this study, we evaluated the adoption of IUS during a 4-year period by TPMG breast surgeons to determine use and efficacy of the new practice.

## Methods

The Research Determination Committee for KPNC determined that the project did not meet the regulatory definition of research involving human subjects per 45 CFR 46.102(d), and an Institutional Review Board review was waived. As an integrated health care system, KPNC has 21 medical centers and more than 4.7 million members. Membership is racially and ethnically diverse and represents the demographics of the northern California population except at the extremes of income.^18^

### Implementation of an Intraoperative US Program

In 2015, as part of an initiative to optimize subspecialty consistency and outcomes, TPMG established the Breast Surgery Group.^19^ The Breast Surgery Group treats more than 4000 new breast cancer patients annually and has consolidated breast operations to high-volume surgeons who perform more than 50 breast operations per year and are committed to incorporating new evidence-based guidelines and techniques into their practices.

To optimize the quality and consistency of KPNC breast care further, the Breast Surgery Group developed a process that consists of initiating pilot projects at one or a few facilities, sharing findings with the remainder of the Breast Surgery Group, and then implementing best practices throughout KPNC. Both the KPNC Mastectomy Surgical Home Recovery program^20,21^ and the adoption of nonopioid postoperative regimens^19^ are successful examples of this process.

The IUS program was initiated in 2015 with a 10 month, four-facility pilot project (1 January to 31 October 2015). The project evaluated the feasibility of using IUS instead of wire-localization for nonpalpable, US-visible breast lesions, with larger goals of improving patient experience and operational efficiency.^17^ After determining that wire localizations could be decreased by 80% with similar re-excision rates and lumpectomy volumes, IUS was promoted as the most patient-friendly and operationally efficient method for locating breast lesions. Breast imagers across KPNC were requested to place US-visible clips into all lesions biopsied under US guidance and routinely include the o’clock position and distance from the nipple in their imaging reports to help orient surgeons and enable them to perform IUS.

In addition, during breast cancer case conferences, breast imagers and surgeons reviewed the US images of patients with new diagnoses and indicated which patients were good candidates for IUS. The pilot-site surgeons created a tip sheet to assist breast surgeons in learning IUS (Fig. S1). Suggestions included performing US in both the office and the operating room on all patients with superficial tumors larger than 1 cm to develop comfort and confidence with US visualization. To transition from wire localization to IUS, a stepwise sequence was recommended, starting with performing intraoperative US on US-visible tumors that had been wire-localized. This provided the security of the wire to guide surgical excision while enabling surgeons to develop confidence with US.

When surgeons became comfortable visualizing wire-localized, US-visible lesions, the next stage was to request that breast imagers mark the skin overlying the tumor before surgery instead of placing a wire. When surgeons developed enough experience with skin-marked tumors, they progressed to IUS without a wire or skin marking. These practices helped avoid missing the tumor intraoperatively during the learning period.

Discussions about IUS implementation were held during quarterly breast surgery leader meetings. Meanwhile, IUS champions within the Breast Surgery Group helped train other breast surgeons, both within and outside of their medical centers. Breast surgeons also were encouraged to take training courses. The Breast Surgery Group did not provide formal training courses, nor did it stipulate what type of training or how much training each surgeon needed to complete. Ultrasound units from 2016 onward were purchased for breast surgeons as needed by the KPNC Clinical Technology team, which develops contracts with vendors and purchases equipment for all KPNC facilities. Additional units were purchased by local facilities.

### Clinical Data

We identified breast cancer patients who underwent breast-conserving surgery (BCS) during the pilot project period (1 January to 31 October 2015) and patients with BCS between 1 January and 31 October 2019 using the KPNC Breast Cancer Tracking Service (BCTS), which maintains a prospective database of all KPNC breast cancer patients. We included patients with a history of breast cancer, those who had undergone neoadjuvant chemotherapy, and those with oncoplastic procedures. Before 2018, we did not have distinct operating room procedure codes to differentiate between localization types, nor were non-radioactive seed localizations available in KPNC.

The 2015 pilot project cohort provided the most accurate representation of IUS cases before 2018. In 2018, non-radioactive seeds became available in KPNC, and by 2019, operating room procedure codes had been established for IUS as well as for seed and wire localization, enabling reliable capture of these localization procedures. Cohort clinicopathologic characteristics were collected with KPNC HealthConnect electronic medical record (EMR) databases (Epic, Verona, WI, USA). The datapoints were age, race, body mass index (BMI), American Society of Anesthesiologists (ASA) physical status score, breast density, whether the patient’s diagnosis was determined after screening mammography or after a diagnostic exam for breast symptoms, history of breast cancer, receipt of neoadjuvant chemotherapy, and type of localization. Tumor characteristics included histologic type, tumor size, and receptor status.

Outcomes included the extent of IUS utilization by individual surgeons. Other outcomes were time intervals on the day of surgery, with a focus on the time from admission to the time of incision; re-excision rates; and whether patients were able to continue with breast conservation or required a completion mastectomy.

### Statistical Analysis

Clinicopathologic variables were summarized, and comparisons were made between the localization techniques using analysis of variance (ANOVA) for continuous variables and Pearson chi-square for categorical variables, with statistical significance defined as a *p* value lower than 0.05. A multivariable logistic regression was used to determine independent predictors of re-excision and examine effects of patient and tumor characteristics, surgery, and localization type. We included all patients in 2019 with a localization procedure except for those with missing values.

All the variables were placed into the model. Symptomatic presentation, BMI, and history of breast cancer had *p* values higher than 0.1 in univariate regression and were not included in the multivariable analysis. All significant and clinically relevant variables were reported as significant if *p* was lower than 0.005 in the final model. All 230 statistical analyses were performed using SPSS Statistics version 22 (IBM, Armonk, NY, USA).

## Results

**Fig 1:**
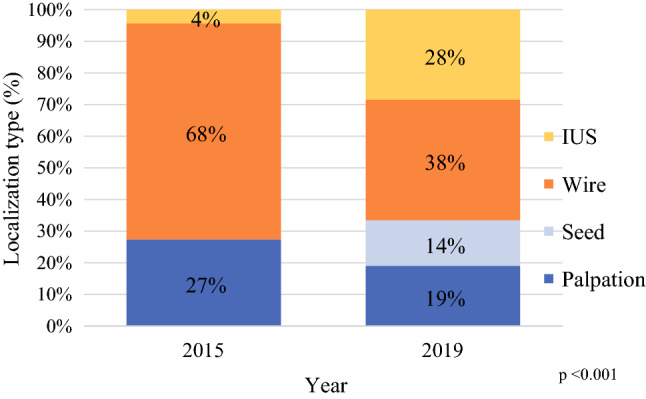
Distribution of lumpectomy types for breast-conserving therapy in 2015 (*n* = 1815) versus 2019 (*n* = 2226)

**Fig 2:**
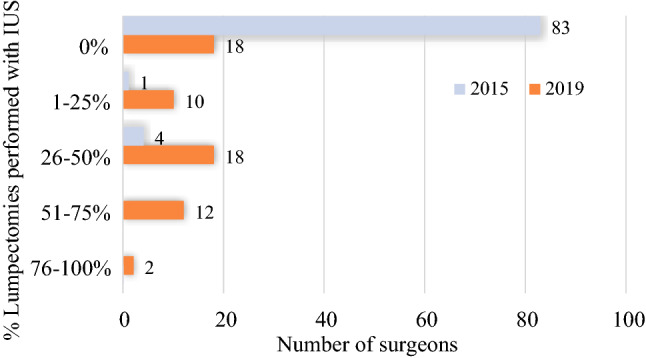
Distribution of surgeon intraoperative ultrasound use (IUS) for lumpectomy in 2015 (*n* = 87) versus 2019 (*n* = 60)

The number of breast-conserving operations increased 23%, from 1815 procedures in 2015 to 2226 procedures in 2019, and IUS lumpectomies increased from 4% to 28% of all lumpectomies (*p* <0.001; Fig. 1). In parallel, breast surgeon utilization of IUS increased significantly (Fig. 2). The total number of breast cancer surgeons decreased from 88 in 2015 to 60 in 2019, consistent with the breast surgery consolidation initiative. Whereas only 5 pilot project surgeons used IUS for breast cancer in 2015 (5 of 88 total breast surgeons, 6%), 42 (70%) of 60 surgeons used IUS in 2019.

We evaluated the distribution of IUS utilization among IUS surgeons and found that 33% of surgeons (*n* = 14) used IUS for 50–100% of their localized lumpectomies, whereas 43% (*n* = 18) used IUS for 25–50% and 24% (*n* = 10) used IUS for 1–25% of these operations. Of the surgeons who adopted US in 2019, the total breast case volume in 2019 did not differ significantly from that of non-adopters (median, 34.5 vs 28 cases; *p* = 0.282), thus total surgeon operative volume did not appear to affect the decision to adopt IUS.

We compared the characteristics of the 2019 cohort by localization type (Table 1) after maturation of the IUS pilot program. History of breast cancer, BMI, and receipt of neoadjuvant chemotherapy were similar between the groups. The IUS cohort had a significantly higher percentage of patients older than 65 years at the time of diagnosis than the groups using other localization techniques (IUS, 54%; wire, 46%; seed, 37%; *p* < 0.001) and a higher percentage of White patients (IUS, 61%; wire, 55%; seed, 47%; *p* < 0.001; *p* < 0.001). The localization cohorts also showed significant differences in breast density (*p* < 0.001). The percentage of patients with an ASA score above 2 was higher in the IUS cohort (IUS, 35% vs wire, 32% and seed, 26%; *p* = 0.013). A higher percentage of patients in the IUS group presented with symptomatic disease (IUS, 40%; wire, 18%; seed, 20%; *p* < 0.001). Notably, the IUS group had a significantly lower percentage of patients with ductal carcinoma in situ (DCIS) (IUS, 7%; wire, 28%; seed, 22%) and a higher percentage of patients with T2 tumors (IUS, 29; wire, 17%; seed, 17%) and invasive ductal carcinomas (IDC) (IUS, 85; wire, 65%; seed, 72%; all *p* < 0.001). Finally, the IUS group had the highest percentage of hormone receptor-positive and **human epidermal growth factor receptor** 2 (HER2)-negative breast cancers (IUS, 83%; wire, 74%; seed, 78%; *p* < 0.001).

**Table 1:** Cohort characteristics by localization technique, 2019

	Localization technique (total *n* = 1803)				
Characteristic	Wire (*n* = 849) *n* (%)	Seed (*n* = 322) *n* (%)	Ultrasound (*n* = 632) *n* (%)	*p* Value	
Age (years)					
<40 40–65 >65	13 (2) 450 (53) 386 (46)	9 (3) 195 (61) 118 (37)	17 (3) 275 (44) 340 (54)	**< 0.001**	
Race					
White Asian/Pacific Islander Black Hispanic Other/multiple/unknown	466 (55) 128 (15) 82 (10) 129 (15) 44 (5)	150 (47) 90 (28) 15 (5) 55 (17) 12 (4)	388 (61) 98 (16) 41 (7) 72 (11) 33 (5)	**< 0.001**	
BMI ≥ 30 kg/m^2^	351 (41)	125 (39)	226 (36)	0.093	
ASA > 2	274 (32)	82 (26)	220 (35)	**0.013**	
Breast density					
1 2 3 4	21 (3) 448 (53) 362 (43) 16 (2)	27 (8) 148 (46) 133 (41) 14 (4)	26 (4) 333 (53) 243 (38) 30 (5)	**< 0.001**	
Detection method					
Screening mammogram Symptomatic	693 (82) 155 (18)	259 (80) 63 (20)	378 (60) 254 (40)	**< 0.001**	
History of breast cancer	53 (6)	10 (3)	41 (7)	0.076	
Neoadjuvant chemotherapy	52 (6)	15 (5)	29 (5)	0.360	
T stage					
Tis T1 T2 T3	237 (28) 456 (54) 142 (17) 14 (2)	73 (22) 192 (60) 55 (17) 1 (1)	46 (7) 394 (62) 185 (29) 7 (1)	**< 0.001**	
Histology					
IDC ILC DCIS	551 (65) 59 (7) 237 (28)	231 (72) 19 (6) 72 (22)	537 (85) 53 (8) 42 (7)	**< 0.001**	
Receptor status^a^					
HR+/HER2– HR+/HER2+ HR–/HER2+ HR–/HER2–	450 (74) 77 (13) 29 (5) 50 (8)	194 (78) 23 (9) 9 (4) 17 (7)	487 (83) 32 (6) 15 (3) 49 (8)	**< 0.001**	

BMI, body mass index; ASA, American Society of Anesthesiologists status; IDC, invasive ductal carcinoma; ILC, invasive lobular carcinoma; DCIS, ductal carcinoma in situ; HR, hormone receptor; HER2, human epidermal growth factor receptor 2

^a^Invasive cancer only

We evaluated the impact of localization type on different time intervals during the day of surgery (Table 2). The time from admission to incision was significantly lower for IUS and seed localizations than for wire (mean, 203 min for IUS, 201 min for seed, and 251 min for wire; *p* < 0.001). In contrast, the time from incision to closure was significantly longer for IUS and seed than for wire (mean, 80 min for IUS, 78 min for seed, and 72 min for wire; *p* < 0.001).

**Table 2:** Day of surgery time intervals by localization technique in 2019

Time interval	Localization technique			
	Wire (*n* = 849) *n* (95% CI)	Seed (*n* = 322) *n* (95% CI)	Intraoperative ultrasound (*n* = 632) *n* (95% CI)	*p* Value
Mean time from admission to incision (min)	251 (246–257)	201 (193–210)	203 (197–209)	**<0.001**
Mean time from incision to closure (min)	72 (69–74)	78 (73–82)	80 (77–84)	**<0.001**
Lumpectomy	50 (47–53)	56 (50–63)	55 (50–60)	0.116
Lumpectomy/sentinel node	78 (75–81)	79 (75–84)	83 (79–86)	0.154
Lumpectomy/axillary dissection	103 (91–115)	122 (99–147)	108 (100–118)	0.270
Mean time from admission to discharge (h)^a^	8.1 (7.8–8.3)	7.0 (6.6–7.4)	7.4 (6.8–8.0)	**0.005**
Lumpectomy	7.0 (6.8–7.3)	5.5 (5.2–5.9)	5.8 (5.3–6.2)	**<0.001**
Lumpectomy/sentinel node	8.4 (8.0–8.7)	7.5 (6.9–8.1)	7.4 (6.9–7.9)	**0.003**
Lumpectomy/axillary dissection	8.3 (7.8–8.8)	7.8 (7.1–8.6)	7.8 (7.3–8.3)	0.373

CI, confidence interval

^a^Only in patients who were discharged same day as ambulatory surgery

When we stratified by operation (lumpectomy alone, lumpectomy with sentinel node biopsy, and lumpectomy with axillary dissection), we found similar trends with longer times for IUS and seed than for wire for each operation type, but these trends did not reach statistical significance. Despite the longer incision to closure times, the length of hospital stay from admission to discharge was significantly shorter for the IUS and seed patients than for the wire patients (7.4 h for IUS, 7.0 h for seed, and 8.1 h for wire; *p* < 0.001). The surgery day was significantly shorter for the lumpectomy patients and lumpectomy with sentinel node biopsy patients who had IUS or seed, and also shorter for the lumpectomy with axillary node dissection patients, although the difference did not reach statistical significance for the latter group.

**Fig 3:**
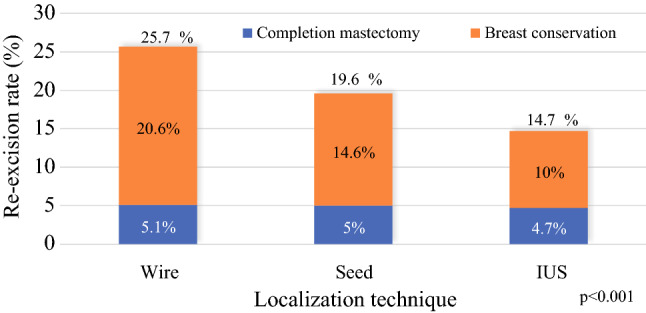
Re-excision rates by localization type in 2019

Finally, we evaluated differences in re-excision rates between the localization types (Fig. 3). The IUS group had the lowest re-excision rate (14.7% of all IUS lumpectomies vs 25.7% of the wire group and 19.6% of the seed group; *p* < 0.001). The need for completion mastectomy was similar between the groups (4.7% of all IUS lumpectomies vs 5.1 of the wire group and 5% of the seed group; *p* = 0.17). The IUS margin re-excision rates for lobular carcinoma and DCIS were 32.1% (*n* = 53) and 26.2% (*n* = 42) respectively, both significantly higher than the 12.1% rate for IDC (*n* = 537) (*p* < 0.001).

The number of IUS cases per surgeon who adopted IUS ranged from 1–58 cases/surgeon in 2019, with a median of 13 cases. We evaluated the re-excision rate for each surgeon stratified by a case volume above or below the median of 13 cases. Those in the top 50% of IUS case volume had a mean re-excision rate of 12.5% (95% CI 8.4–16.6%), which was significantly lower than the mean re-excision rate of 20.6% for those in the bottom 50% (95% CI 8.6–32.6%).

We performed a multivariable logistic regression to determine predictors of re-excision (Table 3). With this model, the risk of re-excision appeared to be lower for older patients, those receiving neoadjuvant chemotherapy, surgeons with a higher operative volume, and patients with hormone receptor-positive status. The risk was higher for those with larger tumors or DCIS and lobular histology. After controlling for confounders, IUS was associated with a lower risk of re-excision (odds ratio [OR], 0.59; 95% CI 0.44–0.80; *p* < 0.001) than wire localization.

**Table 3:** Predictors of re-excision using a multivariable logistic regression model for all patients (*n* = 1799) with a localization procedure in 2019

	OR (95% CI)	*p* Value
Age per year	0.99 (0.98–0.99)	**0.030**
Neoadjuvant chemotherapy	0.14 (0.06–0.34)	**< 0.001**
Surgeon operative volume per case	0.99 (0.98–0.997)	**0.002**
Surgery type		
BCS BCS+SLNB BCS+ALND	Ref 1.71 (1.03–2.83) 3.56 (1.94–6.49)	
		**0.038**
		**< 0.001**
Histology		
DCIS and IDC Lobular carcinoma	Ref 1.87 (1.22–2.88)	
		**0.004**
Hormone receptor positive	0.57 (0.40–0.81)	**0.002**
T stage		
T1	Ref	
T2	1.46 (1.05–2.01)	**0.023**
T3	2.13 (0.70–6.49)	0.185
DCIS	3.18 (1.90–5.34)	**< 0.001**
Localization type		
Wire	Ref	
Seed	0.82 (0.58–1.15)	0.254
Ultrasound	0.59 (0.44–0.80)	**< 0.001**

The model accounts for age, BMI, ASA class, history of breast cancer, neoadjuvant chemotherapy, localization type, method of diagnosis, breast density, surgeon volume per year, tumor hormone receptor positivity, histology, index surgery type, and T stage, and only significant variables are shown.

OR, odds ratio; CI, confidence interval; BCS, breast-conserving surgery; SLNB, sentinel lymph node biopsy; ALND, axillary lymph node dissection; DCIS, ductal carcinoma *in situ;* IDC, invasive ductal carcinoma; BMI, body mass index; ASA, American Society of Anesthesiology

## Discussion

This report describes the implementation of IUS for breast-conserving surgery in a large, integrated health care system. During a period of 5 years, the percentage of IUS lumpectomies increased from 4 to 28% of all breast cancer lumpectomies. In 2019, 70% of breast surgeons used IUS. The average length of the surgery day was significantly shorter with IUS than with wire localization, although operations with IUS required more time than wire-localization operations. The re-excision rates were lower for IUS than for any other localization technique. Overall, the implementation of IUS across our health system has been successful and effective.

Previous studies have described single-institution experiences with small numbers of surgeons^22,23^ or pilot studies with only a few centers.^17^ In contrast, our study describes the largest reported cohort of IUS patients (*n* = 632) and surgeons (*n* = 42). Consistent with our findings, prior studies have compared IUS to wire localization and found significantly lower rates of re-excision with IUS.^1,24–26^ However, our study expanded on previous studies by examining an additional method of localization (non-radioactive seeds), evaluating the impact of localization type on time intervals on the day of surgery and evaluating predictors of re-excision.

Notably, the IUS program developed largely through voluntary adoption of this technique by breast surgeons throughout Northern California in partnership with their local breast-imaging teams. In contrast to other studies, this study had no standardized protocols, formal training, or defined patient selection criteria. Tumor size, depth in the breast, breast size, and density were not strictly used as criteria for IUS, but ability to visualize the clip and/or mass in the office at the initial consultation by the surgeon was required.

The decisions about speed of progression through the steps to independent IUS and candidacy for IUS were at the discretion of individual surgeons. Utilization of IUS was based on surgeons’ confidence as they gained experience with the technique. The distribution of IUS utilization among surgeons in 2019 may reflect differences in level of experience. More experienced surgeons may have had the confidence to use IUS rather than wire or seed localization for a larger percentage of their lumpectomies. Adoption of IUS did not appear to be related to breast surgeon case volume, and may have been driven more by non-surgeon-specific factors (e.g., breast radiologist clip placement, equipment availability, US training).

The majority of IUS patients had tumors easily visible with US, most IUS patients (92%) had T1 or T2 tumors, and 85% had IDC. We hypothesize that some surgeons used IUS in conjunction with palpation, which may have accounted for the decrease in palpation-guided lumpectomies from 27% in 2015 to 19% in 2019. In addition, IUS may have helped improve re-excision rates for these tumors, similar to a report by investigators who conducted a randomized prospective trial evaluating the use of IUS for palpable lesions.^25^

In contrast to the patients with IDC and T1 or T2 tumors who comprised the majority of the IUS cohort, only 7% of the IUS patients had DCIS. Furthermore, only 28% of all the DCIS patients underwent IUS, whereas the remaining 82% had wire or seed localizations. These findings likely reflect the fact that most DCIS patients present with calcifications, which typically are not US-visible, although US-visible clips are placed after stereotactic biopsies at some KPNC facilities. We anticipate that as more breast imagers place US-visible clips for stereotactic biopsy of calcifications and more surgeons become comfortable visualizing the clips and smaller tumors, more patients with small tumors or DCIS will become candidates for IUS.

The magnitude of the difference in re-excision rates between IUS and the other techniques was unexpected (14.7% vs 25.7% for wire and 19.6% for seed localization; *p* < 0.001). We hypothesized that selection bias may have contributed to the low IUS re-excision rates because 85% of the IUS patients had IDC, while only 8% had ILC and 7% had DCIS. With IDC, which typically presents with a well-defined tumor, clear surgical margins are easier to obtain than with the more diffuse ILC and DCIS patterns.^27^ Furthermore, a larger percentage of IUS patients presented with symptomatic tumors and T2 tumors. The ability to palpate the disease during the lumpectomy probably improved the rates of successful surgical clearance. Despite selection bias, the fact that re-excision rates were not worse with IUS than with other techniques and the finding that IUS was a predictor of low re-excision rates in the multivariable analysis suggest that IUS is an oncologically safe alternative to wire and seed localization techniques.

This study had several limitations. The first was the retrospective nature of the study and patient selection biases, as discussed earlier. Second, without standard training methods or tracking, we were unable to determine the number of cases to transition from standard palpation or wire localization to US. Further study is underway to develop a standardized US training program and to measure outcomes of this training.

Finally, although we found differences in time intervals on the day of surgery, we did not capture patient-reported outcomes (PROs) to assess whether levels of anxiety and overall levels of satisfaction differed between the different localization types. We also lacked PROs regarding pain associated with localization and/or the breast operation or satisfaction with aesthetic outcomes. The Breast Surgery Group currently is developing and testing PRO tools to capture these data for future studies.

In conclusion, implementation of an IUS localization program is efficient and feasible for a large, high-volume, multiple medical center practice. Adoption of an IUS localization program required coordinated system changes between surgeons and breast imagers, resulting in significant increases in utilization of IUS over time. The benefits of IUS include avoiding an additional invasive procedure, freeing up breast-imaging department time and personnel to perform other services, and decreasing the length of stay on the day of surgery. Intraoperative ultrasound can be used for the majority of breast cancers, and in this study resulted in lower re-excision than wire or seed localization. We will continue to evaluate how IUS improves patient satisfaction and operative outcomes and anticipate expanding its utilization as surgeons mature in their IUS practices.

## Supplementary Information

Below is the link to the electronic supplementary material.

Tip Sheet for starting an intraoperative ultrasound (IUS) localization program. Supplementary file1 (DOCX 16 kb)
