# Supplementary material for: Implementation of Intraoperative Ultrasound Localization for Breast-Conserving Surgery in a Large, Integrated Health Care System is Feasible and Effective
Source: Ann Surg Oncol. 2021 Aug 26;28(10):5648–56. doi: 10.1245/s10434-021-10454-8 (PMC8418593; doi:10.1245/s10434-021-10454-8)
Supplement: Supplementary file 1 — Tip Sheet for starting an intraoperative ultrasound (IUS) localization program. Supplementary file1 (DOCX 16 kb) [file 10434_2021_10454_MOESM1_ESM.docx]

**Supplemental Figure 1.** Tip Sheet for starting an intraoperative ultrasound (IUS) localization program.

Obtain ultrasound machines for both clinic and the operating room (OR)

- In-service from company representative
- Determine which probes to use and purchase – both in clinic and the OR

Learn ultrasound skills

- Learn most utilized buttons and knobs
- Orientation of probe and images on screen.
- Understand imaging terms “gain, depth, axis.”

Service agreement with breast imagers

- Place ultrasound visible clip at time of mammogram or US guided core needle bx (CNB)
- Standardize breast imaging reports to include location: x o’clock, y cm from nipple.
- Check availability for breast imagers in case you need help in the OR

Surgeon Utilization

- Decide which surgeons want to do IUS localization and send appropriate referrals to these surgeons or have a department “champion.”
- Privileging - Should not need to request new privileges

Create Ultrasound Workflow

- Perform ultrasound as part of the breast exam. If you can see clip, schedule for the OR. If not, schedule for skin marking or wire localization
- Ultrasound patient in pre-op area to confirm clip and location
- Specimen mammogram to document clip/mass removal

Standardize documentation in medical record

- Smartphrase which captures key portions of ultrasound exam and intraoperative components – Include: clip seen. Mass/lesion seen

Compile a database of patients and outcomes

- Utilize support staff to compile patient records for future analysis of outcomes

**PITFALLS**

- Radiologist unable to do skin marking – schedule as needle localization
- Clip not in specimen on mammogram – missing clip
  - Pre-op discussion with patient
  - Await path results: you may still have removed the lesion of concern, biopsy site changes
  - Post-operative mammogram to assess for residual clip
- Consider the size of lesion – if small and the mass is not easily seen, then do not rely on ultrasound alone

**PEARLS**

- Easier to see lesion and clip if at least 5 days from CNB.
- Start with reviewing ultrasound images w/ breast radiologists at breast cancer case conference.
- Practice ultrasound with other providers (anesthesia) in the OR to gain more familiarity
- Optimize patient positioning in OR (oblique position for lateral lesions)
- Probe cord – fix around your neck or to OR drapes to lessen tugging on the probe and maximize accuracy
- Marking skin – In OR on the skin before starting the operation (tic tac toe grid or box)
- Look at specimen mammogram to decrease re-excision rate, look at intraoperative margins after excised
